# The impact of lockdown on child adjustment: a propensity score matched analysis

**DOI:** 10.1186/s40359-024-01894-4

**Published:** 2024-07-27

**Authors:** Wanjuan Weng, Mowei Liu, Shumin Wang, Xiaoyun Li, Jinghui Zhang, Yuke Fu, Chuanmei Dong, Yan Li

**Affiliations:** 1 Shanghai Institute of Early Childhood Education, Shanghai Normal University, 100 Guilin Road, Xuhui District, Shanghai, China; 2 Department of Psychology, Trent University, Peterborough, Canada; 3 School of Education, Macquarie University, Sydney, Australia

**Keywords:** COVID-19, Lockdown, Preschooler, Parent–child conflict, Social adjustment

## Abstract

The COVID-19 pandemic has had an inestimable impact worldwide, challenging the daily lives and interactions of children and their families. In 2022, Shanghai implemented a three-month lockdown in response to an acceleration of positive cases during the pandemic period. This restrictive policy provided insight into the impact of the lockdown on children's social adjustment and the role of parent–child conflict during this process. Mothers of preschool-aged children participated in this study and completed the Chinese version of Child-Parent Relationship Scale (CPRS) and the Strengths and Difficulties Questionnaire (SDQ). Using Propensity Score Matching (PSM) method, two matched groups were formed: pre-lockdown group and post-lockdown group, with a total of 574 preschoolers (*N* = 297 in each group; *M*_age_ = 4.36, *SD* = 0.86) were recruited. The results showed that the lockdown directly impacted children's emotional symptoms. Additionally, the parent–child conflict mediated relationship between the lockdown and children's adjustment. Specifically, parent–child conflict deteriorated children's emotional symptoms, hyperactivity-attention problems, and prosocial behaviors. These findings highlight the significant impact of the severe lockdown on children's social adjustment and the role of parent–child interactions during this period.

During the COVID-19 pandemic, many institutions and authorities have implemented essential restrictive measures, such as social distancing measures and lockdowns, to curb its spread [1, 2]. Since March 11, 2022, Shanghai's city-wide lockdown, which includes the closure of all kindergartens, has presented new challenges for affected families [3]. Home confinement during lockdowns has particularly burdened parents of preschoolers with concerns about infection, economic pressure, and caregiving responsibilities [4, 5], depleting their energy and motivation [6], and affecting parenting skills [7] and parent–child relationships [8, 9]. The joint effect of limited social interaction and increased parent–child conflict during lockdowns may manifest in children's emotional distress and problematic behaviors. While existing research supports the deterioration of children's social adjustment during the pandemic [10], most studies lack clarity regarding the unique effect of COVID-19 lockdowns and how their implementation affects parent–child conflict, which, in turn, affects child adjustment [11–13]. The present study adopts a novel approach by utilizing propensity score matching (PSM) (1) to investigate the direct and unique impact of lockdown measures on social adjustment in preschoolers and (2) to explore whether the relationship between lockdown and social adjustment is mediated by parent–child conflict.

## COVID-19 lockdown and child social adjustment

Social adjustment refers to an individual's ability to effectively establish harmonious relationships with others while engaging in satisfactory social interactions [14]. In the context of preschoolers, social adjustment is commonly measured using several key indicators, including social competence, externalizing behavior, and internalizing behavior [15–17]. Previous research has consistently shown that early maladjustment has been found to correlate with poorer academic performance and challenges in adapting to school environments during later stages of development [18–20].

There is little doubt that the global COVID-19 pandemic has altered the daily lives of many children, subsequently, affecting their early learning and development. According to the life course theory, individual development trajectories are affected by interrelated and intertwined stratification systems [21]. It is widely recognized that the pandemic, along with the social changes associated with the lockdowns, serves as a notable social and historical stressor event [22, 23]. The implementation of lockdown measures during the pandemic has resulted in fundamental changes to the daily routines of young children [24]. Liu and colleagues found that extended periods of stay-at-home measures during lockdowns led to more unpredictable events and an increased risk of social maladjustment [12]. The closure of educational settings (schools and kindergartens) due to the pandemic has deprived children of opportunities for social activities and interactions, leading them to increasingly rely on electronic media as alternative ways of maintaining communication and acquiring essential social skills [25, 26]. Lastly, preschool-aged children lack coping strategies and adequate social support to effectively deal with fear and uncertainty during the pandemic [27]. These factors could pose significant developmental risks for social maladjustment among preschoolers during lockdown periods.

Recent research has highlighted the increased psychological and behavioral problems in children during the pandemic [4, 10, 28, 29]. Several empirical studies have supported the notion that young children exhibit notable social maladjustment following the pandemic [11, 30]. For instance, a study involving Italian preschoolers showed that during the lockdown, young children experienced heightened emotional symptoms and behavioral problems [11]. Similarly, in China, school-age children were found to have experienced significant negative impacts on social adjustment, with the incidence of behavioral problems ranging from 4.7% to 10.3%, including conduct problems, peer problems, hyperactivity-inattention, and emotional problems, as a result of the lockdown measures [31].

To our knowledge, limited research has been conducted to explore the impact of lockdowns on the social adjustment of Chinese preschool children. This is an area that requires attention, as the preschool years are crucial for a child's early learning and development, including the acquisition of social adjustment skills [32, 33]. Moreover, China had approximately 480.521 million preschoolers in 2021 [34]. Including Chinese young children as participants expands the scope of international research in early childhood psychology, which has been predominantly by Western research.

## The mediating effect of parent–child conflict

Parent–child conflict is a significant aspect of an unhealthy parent–child relationship. It is characterized by both parents and children engaging in negative behaviors, such as discordant or acrimonious interactions [35]. According to Bronfenbrenner's ecological systems theory, parents, as the immediate environment in which children grow up (i.e. microsystem), directly influence the development of children's adjustment [36]. Early instances of discord and conflict between parents and children may lead to the development of dysfunctional patterns of interaction [37], ultimately having a detrimental impact on a child's display of externalizing and internalizing problems [38–40]. For example, previous research suggests that children exposed to parent–child conflict tend to experience a more negative family atmosphere, which would, in turn, increases the probability of problematic behavior in children [41–43].

The negative impact of parent–child conflict on children's social adjustment may become more devastating during the lockdown period. From the perspective of family stress theory (FSM), the pandemic can be viewed as a source of stress, a non-normative life event with the potential to disrupt the social dynamics within a family system [35, 44, 45]. Recent empirical studies have revealed that the increase in parent–child conflict during the pandemic can be attributed to disruptions in daily life routines and the overall chaotic family environment [12, 46]. Prime and colleagues [9] explained that the effects of the pandemic permeate various aspects of family functioning, including parent–child relationships, thereby posing significant risks to children's adjustment.

Indeed, during the lockdown, parents experienced high levels of stress and feelings of being overwhelmed due to multiple factors, including the economic crisis, the risk of infection, work-related issues such as job loss, excessive workload, and reduced income [4, 5, 47]. Under such circumstances, parents were unable to fully attend to their children's needs or provide high-quality parenting practices [48, 49]. A longitudinal study conducted before and after the pandemic found that parents' psychological well-being deteriorated during the pandemic, as evidenced by decreased self-efficacy and increased psychological distress [50].

As a logical progression, the stress experienced by parents during the lockdown may result in low-quality parent–child interactions and a negative parent–child relationship, which in turn affects the social adjustment of their children [48]. It is worth noting that the parent–child relationship during early childhood influences children's development and behaviors at preschool age [51]. The aforementioned studies primarily examined children across different age groups, with limited focus on preschool children and their parents.

In conclusion, based on the ecological systems theory and the family stress model, the stress resulting from the lockdown may lead to the escalation of parent–child conflict within families. As a consequence, the increased occurrence of parent–child conflicts poses a threat to the healthy development of children's social adjustment. In this study, we aim to investigate the complex relationship between the COVID-19 lockdown, parent–child conflict, and various indicators of social adjustment (including emotional symptoms, hyperactivity-inattention, and prosocial behavior) among preschoolers.

### The present study

Many studies have focused on changes in preschool children's social adjustment or alterations in parent–child relationships during the pandemic. However, few studies have investigated whether lockdown itself is the primary cause of children's social adjustment issues and parent–child conflict. This is mainly due to the lack of conditions necessary to establish control and experimental groups to manipulate the COVID-19 conditions. Therefore, the present study adopted an innovative approach by employing the propensity score matching technique. The use of propensity score matching allowed us to examine the unique contribution of COVID-19 lockdowns to parent–child conflict and children's social adjustment by mimicking the statistical procedure of experimental studies and comparing outcomes before and after the lockdown (e.g., pre-lockdown versus post-lockdown). The participants remained the similar in terms of their socio-demographic background (e.g., child age, child gender, the number of a siblings, mother's educational level, registered permanent residence, and family structure). The present study aimed to shed light on the impact of the COVID-19 lockdowns on social adjustment in Chinese preschoolers and explore the interplay among the lockdowns, parent–child conflict, and social adjustment in children. This study posits two hypotheses: (1) that lockdown has a negative impact on child social adjustment, and (2) that parent–child conflict mediates the relationship between lockdown and child social adjustment. Specifically, the lockdown condition is expected to exacerbate children's social adjustment problems through higher incidences of parent–child conflict.

## Methods

### Procedure

The present study included two distinct groups of participants, the pre-lockdown and the post-lockdown. The participants were mothers who had at least one preschool-aged child attending a local kindergarten in Shanghai. Data collection for both groups took place during the pandemic period. The pre-lockdown (*N* = 900) completed the questionnaires in October 2020. At this time, although the pandemic had begun in December 2019, the situation in Shanghai was relatively stable with only 191 new cases reported throughout the entire month. While certain restrictions were still in place, daily life was not severely disrupted. In contrast, the post-lockdown (*N* = 1249) completed the questionnaires during a city-wide lockdown in 2022. This lockdown was implemented in response to a significant surge in COVID-19 cases, with daily infections reaching up to 20,000 and a total of 100,000 cases reported in April 2022. Lasting for over three months, the lockdown significantly restricted movement, impacting work, school, and daily life. As the data from both groups were collected during the pandemic period, any observed score differences in parent–child conflict and social adjustment between the two groups can be attributed to changes such as the stress induced by the city-wide lockdown rather than the broader effects of the pandemic.

### Propensity score matching

Propensity score matching (PSM) has been widely used in the field of education and evaluation research. It serves as a valuable tool for inferring causality in non-randomized research by addressing differences between two samples using a comprehensive set of covariates [52]. PSM involves a two-step procedure, that is, estimating the propensity scores (PSM), and matching individuals based on these scores. In the context of this study, PSM was adopted to examine the effect of COVID-19 lockdown on parent–child relationships and preschool children's social adjustment, while accounting for potential bias arising from confounding variables (such as socio-demographic characteristics). By employing PSM, this study aimed to eliminate the influence of such confounding factors and estimate the true effect of the specific variable under investigation [53].

### Participants

After implementing the group-matching procedure, the final sample consisted of 574 preschoolers with 297 participants in each group (*M*_age_ = 4.36, *SD* = 0.86). The matching process ensured that the two groups had similar socio-demographic characteristics, including child age, child gender, the number of siblings, registered permanent residence, mother's educational level, and family structure. According to the results of the National Bureau of Statistics of China [54], the sample in this study can be considered representative of families in medium to large cities in China in terms of education level and family structure. A detailed demographic profile of each group can be found in Table 1. Both sampling groups completed two questionnaires: the Parent–Child Relationship questionnaire and the Strengths and Difficulties Questionnaire. The questionnaires were completed by mothers (*M*_*age*_ = 34.74, *SD* = 4.57).

**Table 1:** Demographic Profile of the Study Groups

	Control group		Lockdown group		
	N	%	N	%	
Child age					
< 4 years	53	17.85	51	17.17	χ^2^ (2) = .426, *p* = .808
4–5 years	194	65.32	201	67.68	
> 5 years	50	16.84	45	15.15	
Child gender					
Girls	149	50.2	155	52.19	χ^2^ (1) = .243, *p* = .622
Boys	148	49.8	142	47.81	
The number of a sibling					
Yes	265	89.22	273	91.92	χ^2^ (1) = .296, *p* = .130
No	32	10.77	24	8.08	
**Mother's educational level**					
High school or elementary school	19	6.40	25	8.42	χ^2^ (4) = 2.842, *p* = .585
Junior college	114	38.38	109	36.70	
Bachelor’s degree	164	55.78	163	54.88	
**Registered permanent residence**					
Local household registration	265	89.23	273	91.92	χ^2^ (1) = 1.262, *p* = .261
Non-local household registration	32	10.77	24	8.08	
**Family structure**					
Nuclear family	281	94.61	276	92.93	χ^2^ (1) = .721, *p* = .396
Joint family or other structure	16	5.39	21	7.07	

### Measures

#### The Child-Parent Relationship Scale (CPRS)

Mothers completed the Chinese version of the Child-Parent Relationship Scale [55, 56]. The CPRS is a widely used and validated tool for assessing the relationship between children and parents, exhibiting strong psychometric properties [57]. It consists of three subscales: intimacy, conflict, and dependence, which provide insight into the nature of the relationship between children and their parents [55]. In this study, the conflict subscale (12 items) was used to measure the extent to which parents perceive their relationships with their children to be characterized by negativity. An example item from this subscale is: "Dealing with my child drains my energy". Participants rated each item on a five-point Likert scale, ranging from 1 (very unlikely) to 5 (very likely). Higher scores indicate a higher level of parent–child conflict. In the present study, the Cronbach's alpha for the conflict subscale was α = 0.85, indicating good internal consistency reliability.

#### The Strengths and Difficulties Questionnaire (SDQ)

Mothers completed the Chinese version of the Strengths and Difficulties Questionnaire [58, 59]. The SDQ has been demonstrated to be an effective tool for identifying mental health issues among children and adolescents in China [60]. The SDQ consists of five subscales, each containing five items, resulting in a total of 25 items. Participants rated each item on a three-point Likert scale, ranging from 1 (not true) to 3 (certainly true). The subscales assess different aspects of children’s behavioral and emotional functioning. Some examples of the items include, "often unhappy, down-hearted or tearful" and "often fights with other children or bullies them". The Cronbach's Alpha coefficients for the subscales were as follows: emotional symptoms (α = 0.60), hyperactivity-inattention (α = 0.75), and prosocial behavior (α = 0.70). Conduct problems and peer problems demonstrated low reliability, which has been revealed in other studies [61, 62]. Consequently, these two subscales were eliminated from further analyses. It should be noted that higher scores on the prosocial behavior subscale indicate strengths, while higher scores on the other subscales (emotional symptoms and hyperactivity-inattention) indicate difficulties.

### Data analytical strategy

Statistical analyses were conducted using SPSS version 26. The significance level for all analyses was set at = 0.05. No missing values were present in the dataset. Initially, Propensity Score Matching (PSM) was employed to filter and match the two sets of data. Chi-squared tests were then performed to ensure that there were no significant differences in social demographic variables between the two groups. To examine whether parent–child conflict mediates the relationship between the lockdown and child social adjustment, the PROCESS macro for SPSS was used for mediation analysis.

## Results

### Preliminary analysis

Matched the pre-lockdown group and the post-lockdown group with similar background characteristics (age, gender, registered permanent residence, the number of a siblings, educational level, and family structure) were created using a propensity score matching procedure with the SPSS PS module [63]. The estimation algorithm used was logical regression, and the matching algorithm utilized was nearest matching with a caliper set at 0.20. An overall balance test was conducted to assess the equivalence between the matching groups [64]. The test results indicated no significant differences between the groups. Furthermore, chi-square tests were performed to examine social demographic information, and the results confirmed that there were no significant differences between the two groups after Propensity Score Matching. All indicators had *p*-values greater than 0.05, indicating no significant discrepancies (refer to Table 1 for details).

As the independent variable (study group) is categorical, Spearman correlation was used to analyze the correlations between the independent variable, mediator and outcome variables. Pearson correlation was used for the analysis of other variables. The correlation analysis results are presented in Table 2. The results revealed that the study group has significant positive correlations with both parent–child conflict and emotional symptoms, indicating that parents from the post-lockdown group reported more conflicts with children and faced more intense child emotional problems than parents from the pre-lockdown group. Additionally, there were significant correlations between parent–child conflict and all three indicators of child social adjustment.

**Table 2:** Correlations among Analyzed Variables of Interest

	1	2	3	4	5
1. Study group	1				
2. Parent–child conflict	.09*	1			
3. Child emotional symptoms	.24***	.31***	1		
4. Child hyperactivity-inattention	.04	.27***	.30***	1	
5. Child prosocial behavior	.02	-.30***	-.21***	-.29***	1
M	-	1.85	1.38	1.81	2.68
SD	-	0.60	0.36	0.50	0.35
Range	-	1–4	1–3	1–3	1–3

Study group: 0 = pre-lockdown group; 1 = post-lockdown group

^*^p < .05

^***^p < .001

### Main effect of the lockdown on child social adjustment

To test Hypothesis 1, a linear regression analysis was conducted using SPSS to examine the effect of the lockdown on child social adjustment. As presented in Table 3, the study group was significantly and positively associated with emotional symptoms (*β* = 0.25, *p* < 0.001), indicating that children in the post-lockdown group exhibited greater emotional symptoms compared to those in the pre-lockdown group. However, the study group was not significantly associated with hyperactivity-inattention and prosocial behavior.

**Table 3:** Test Result of Total Effect

	β	SE	t	R^2^	F
Study group → Emotional symptoms	.249	.080	6.256***	.062	39.143***
Study group → Hyperactivity-inattention	.039	.082	.944	.002	.891
Study group → Prosocial behavior	.015	.082	.371	.000	.138

Study group: 0 = pre-lockdown group; 1 = post-lockdown group

^*^p < .05

^***^p < .001

### Mediation analyses

To further investigate whether parent–child conflict mediated the effects of the lockdown on child adjustment, a mediation model was examined (see Table 4). The results indicated that the lockdown had a significant positive effect on parent–child conflict, and parent–child conflict had significant positive effects on all three indicators of child social adjustment. After including the mediator variable, the study group still had a significant positive effect on emotional symptoms, but not on hyperactivity-inattention and prosocial behavior. Overall, the lockdown can impact child social adjustment through the mediating role of parent–child conflict.

**Table 4:** Test Results of the Mediation Model

	Parent–child conflict			**Emotional symptoms**			**Hyperactivity-inattention**			**Prosocial behavior**		
	β	SE	t	β	SE	t	β	SE	t	β	SE	t
Study group	.176	.082	2.150*	.446	.076	5.853***	.033	.079	.422	.078	.079	.985
Parent–child conflict				.294	.038	7.731***	.248	.040	6.220***	-.271	.039	-6.838***
R^2^	.007			.148			.062			.073		
F	4.622*			51.397***			19.822***			23.459***		

Study group: 0 = pre-lockdown group; 1 = post-lockdown group

^*^p < .05

^***^p < .001

The lockdown's influence on child social adjustment was found to be partly mediated by parent–child conflict. Subsequent mediation analyses confirmed the significance of all three mediation paths, as outlined in Table 5. These outcomes provide support for the mediating function of parent–child conflict in the association between the lockdown and child social adjustment.

**Table 5:** Test Results of Mediating Effect (Indirect Effect)

Model path	Effect	SE	95% confidence interval	
			Lower	Upper
Bootstrapped indirect effects				
Study group → (Conflict) → Emotional symptoms	.052	.025	.004	.102
Study group → (Conflict) → Hyperactivity-inattention	.044	.022	.003	.090
Study group → (Conflict) → Prosocial behavior	-.048	.023	-.095	-.005

Study group: 0 = pre-lockdown group; 1 = post-lockdown group

## Discussion

The COVID-19 pandemic and the subsequent implementation of lockdown measures have had a profound and far-reaching impact on both parents and children. While current studies have focused on changes in social adjustment and the parent–child relationship among preschool children during pandemics, there is a notable gap in exploring the specific effect of lockdown measures on children's social adjustment and parent–child conflicts. To address this gap, the present study used propensity score matching (PSM) to match each family in our sample with similar socio-demographic backgrounds, thereby mitigating potential confounding effects in this line of research. Importantly, our study provides new insights into the perspectives of parent–child conflict and children's social adjustment in the context of the COVID-19 lockdown.

As expected, our findings indicate that, after controlling pre-test covariates related to socio-demographic background, the post-lockdown group exhibited higher levels of parent–child conflict compared to the pre-lockdown group. Our results further suggest that, during the city lockdown, increased parent–child conflict within families was associated with a greater degree of social maladjustment among preschoolers. Specifically, these maladjustment indicators encompassed elevated emotional symptoms, hyperactivity-inattention, and diminished prosocial behavior. Overall, our findings suggest that the COVID-19 lockdown may exacerbate the adverse impact of parent–child conflict on children's social adjustment.

### The impact of lockdown on preschooler social adjustment

Overall, our findings demonstrate a significant effect of the COVID-19 lockdown on the social adjustment of preschool children. This aligns with recent research findings that highlight the escalation of behavioral and psychological problems among children during the pandemic [27, 65–67]. Specifically, our study reveals that the pandemic lockdown had a direct impact on children's emotional symptoms, but did not have a significant direct effect on hyperactivity-inattention and prosocial behavior.

From the perspective of life course theory, the COVID-19 lockdown represents a significant social and historical event in the development of children. The lockdown has brought about a combination of adverse effects on children's adjustment development, which may be further exacerbated by by the joint impact of lifestyle changes and psychosocial stress induced by home confinement [68]. Children at a young age, in particular, may face even more challenges during strict lockdown periods [69, 70]. This is because young children are in a sensitive period of neurodevelopment, lacking independence, coping strategies, and being more sensitive to environmental changes [23]. Under such circumstances, children are more likely to experience physical and social isolation, fear, anxiety, and other negative emotions [13, 27]. Our research confirms that the COVID-19 lockdown directly led to an increase in emotional problems among young children.

Interestingly, it was found that the lockdown had no significant main effect on preschoolers' hyperactivity-inattention. Hyperactivity-inattention refers to difficulties in maintaining attention and controlling one's activity level [71]. Children with hyperactivity-inattention may display excessive motor activity, struggle to stay focused on tasks, and exhibit impulsive behaviors. Because hyperactivity-inattention is primarily associated with attentional control and activity regulation, it is more likely to be perceived as problematic in structured settings such as classrooms, where children are required to follow instructions, complete tasks, and adhere to specific routines [72]. However, preschoolers' academic demands and structured learning activities are generally less intensive compared to school-age children. They may not have encountered significant academic challenges during the lockdown, which could have contributed to the lack of a main effect of the COVID-19 lockdown on hyperactivity-inattention.

Similarly, our findings indicate that the lockdown did not have a direct impact on preschoolers' prosocial behaviors. Prosocial behaviors of preschoolers are commonly observed during interactions with parents and peers, such as sharing toys, offering assistance, and forming relationships [73]. Despite the limitations imposed by the lockdown, children were still able to engage in prosocial behaviors within the confines of their family environment, where interactions with parents and siblings persisted. Consequently, the lockdown did not directly influence preschoolers' prosocial behaviors.

### The mediating effect of parent–child conflict

Our results indicated that the COVID-19 lockdown is associated with higher levels of parent–child conflict among parents of young children in China. This hypothesis is supported by a previous study that demonstrated a link between living in a more unstable situation during the lockdown and a lower-quality parent–child relationship [49]. Overall, the relationship between parents and their children was highly deteriorated during the pandemic [74–76]. The results from this study further provide evidence to the conceptual framework proposed by Prime [9], which suggests that the COVID-19 pandemic, as a social disruption, permeates into the family system. Specifically, changes in lifestyle during the lockdown placed increased stress on parents, particularly those with young children [77]. For instance, parents had to invest more time in protecting their children from the virus and explaining why certain daily activities were canceled. This, in turn, contributed to higher levels of parenting exhaustion and tension in the parent–child relationship [77–79].

As expected, our results also indicate that parent–child conflict has an impact on children's social adjustment. Parent–child conflict generates distress and insecurity in children, which, in turn, affects their social adjustment. This finding was consistent with previous studies that have identified parent–child conflict as a predictor of children's maladjustment [41, 80]. Research focused on children's mental health has shown that, when children feel unsafe during the pandemic, they are more likely to develop psychological and emotional problems, such as increased anxiety and depression [24, 81]. These maladaptive outcomes can have negative implications for children's psychological and behavioral adjustment [82].

Results from the mediation analyses indicated that the COVID-19 lockdown indirectly predicted child maladjustment through its association with parent–child conflict. During the lockdown, children were unable to have contact with individuals outside their immediate family, such as teachers, extended family members, and friends. Parents became the sole providers for their children's physical, emotional, and educational needs [75]. Under such circumstances, parents had to take care of their young children while managing their own daily responsibilities and work, which increased their level of exhaustion, and may lead to changes in their emotional state, parenting stress and parenting style [4, 7, 83]. As a result, the increase in parent–child conflict during the pandemic was associated with higher levels of parental stress [31, 46], which can make parents less attuned to their children's needs and engaged in more dysfunctional parent–child interactions, leading to increased conflict in the parent–child relationship [82]. Consequently, this can contribute to the occurrence of child social maladjustment [4].

Notably, our findings revealed that parent–child conflict played a complete mediating role between the COVID-19 lockdown and both hyperactivity-inattention and prosocial behaviors in preschoolers. In other words, the connections between the COVID-19 lockdown and these two outcomes were indirect, and entirely mediated by the presence of parent–child conflict. These findings further highlight the significance of the quality of parent–child interactions during the lockdown period [84].

The home environment and the support provided by caregivers play a crucial role in managing children's behaviors [85]. With children learning from home during the lockdown, parents had to take on a more active role in monitoring children's behaviors, facilitating their education, and nurturing and modeling prosocial behaviors [86]. The increased stress and disruptions caused by the lockdown can contribute to higher levels of parent–child conflict within the home environment [87]. This conflict may disrupt the child's sense of security, trust and emotional well-being [81, 88]. As a result, children may exhibit symptoms of hyperactivity-inattention, struggling to regulate their emotions and behaviors in response to the conflictual atmosphere. The heightened emotional arousal caused by conflict can make it challenging for children to concentrate, stay focused on tasks, and control their activity level, ultimately leading to an increase in hyperactivity-inattention problems [89]. In addition, conflictual interactions during parent–child interactions may involve negative and coercive parenting strategies, such as criticism, yelling, or punishment [90]. These negative interactions can potentially have a detrimental impact on the child's development of prosocial behaviors. Children may become less motivated to engage in behaviors such as helping, sharing, or cooperating due to the negative experiences associated with conflictual parent–child interactions. The disruption in the parent–child relationship may hinder the child's inclination and ability to exhibit prosocial behaviors [84].

In summary, the increased stress and disruptions caused by the lockdown can result in elevated parent–child conflict, which in turn affects social development in preschoolers. The negative effects of such parent–child conflict on various aspects of social adjustment highlight the importance of addressing and mitigating parent–child conflict, particularly during challenging circumstances such as lockdown.

### Limitations and future directions

The present study employed Propensity Score Matching (PSM) to investigate how the COVID-19 lockdown influenced the social adjustment of preschool children in China. The mediating role of parent–child conflict in the relationship between the lockdown and child adjustment outcomes was also examined. Several limitations that should be acknowledged. First, the study was a cross-sectional survey, which could not establish the temporal sequence of the variable. We could not rule out the reverse causality, wherein child adjustment problems could also contribute to greater parent–child conflict. Secondly, the use of questionnaires during the COVID-19 pandemic limited the study to only collecting data from mothers of young children, which may introduce bias as a single source of data. Future research could use multi-source evaluation methods to obtain a more comprehensive understanding of the role of parent–child relationships in children's social adjustment in the context. Third, this study primarily focused on the impact of the parent–child relationship on children's social adjustment. Other factors such as parental stress and coparenting has proven to be strong predictors of externalizing behavior in young children [78]. Given the enormous stress experienced during the COVID-19 pandemic, exploring the protective role of various caregiver supports (e.g. social support, economic support, marital quality, and positive coping strategies) on children's adjustment would be valuable for future studies.

Finally, while we controlled for the participant's age, gender, mother's educational background and family structure, we did not account for family economic status and regional differences. It is worth noting that low-income families may face additional challenges due to limited resources and rising prices, potentially increasing the risk of children's exposure to the virus [91, 92]. Moreover, our research sample was collected from Shanghai, China, where the average education level and income may be higher than in other regions. This may not be representative of the entire nation. Future research should aim to replicate this study in diverse family backgrounds and regions to enhance the generalizability of the findings.

## Conclusion

While previous research has sought to estimate the negative impact of COVID-19 on children and the parent–child relationship, the pandemic itself is often regarded as a research context rather than a variable to be manipulated or analyzed. This study conducted a direct comparison between the post-lockdown group and the pre-lockdown group to examine the impact of the COVID-19 lockdown on children's social adjustment and parent–child conflict. The findings suggest that the lockdown contributed to increased parent–child conflict and had a direct impact on various social adjustment indices in children. These findings highlight the negative impact of the lockdown, particularly in relation to parent–child conflict, on the social adjustment of young children. These findings have important implications for early intervention and educational programs that aim to enhance the well-being and adjustment of young children. In adverse situations, it is crucial to prioritize and address the parent–child relationship within families to provide necessary assistance and support.

## Data Availability

The datasets used and analysed during the current study are available from the corresponding author upon reasonable request.
